# Protection of multiple aspects of Alzheimer’s disease pathology using dietary supplementation with taurine

**DOI:** 10.21203/rs.3.rs-7483320/v1

**Published:** 2025-10-06

**Authors:** Christina M. Tognoni, Rajshree Ghosh Biswas, Zeynep Melis Suar, Isabel Carreras, Alpaslan Dedeoglu, Bruce G. Jenkins

**Affiliations:** VA Boston Healthcare System; Harvard Medical School; Harvard Medical School; VA Boston Healthcare System; VA Boston Healthcare System; Harvard Medical School

**Keywords:** Alzheimer’s Disease, Taurine, Magnetic resonance spectroscopy, metabolomics, preventative measures

## Abstract

As Alzheimer’s disease (AD) continues to rise amongst the aging population, preventative measures such as dietary or lifestyle changes represent an attractive option to mitigate the burden. Taurine, known for its antioxidant and anti-inflammatory properties, may also play a neuroprotective role. This study investigates the protective effects of taurine supplementation in 5xFAD mice. Taurine was administered through drinking water at doses of 0, 500, 1000, 2000, 4000 mg/kg/day, with no change in water consumption or body mass was observed. Postmortem markers of neuroinflammation using cytokine profiling demonstrated that 2000 mg/kg/day was effective at invoking a protective response against AD progression. An acute dose of this concentration, in older mice, was also sufficient at protecting the dentate gyrus against gliosis and preventing volume loss. Supplementation of taurine for 1–2 months in older mice also led to a small, reduction in the Aβ42 burden. This suggests that both long-term and acute administration of taurine can mitigate pathological characteristics of AD. High-resolution magic angle spinning magnetic resonance spectroscopy (HRMAS-MRS) was used to analyze and differentiate the molecular profile of 3 key AD-affected regions: frontal cortex, ventral and dorsal hippocampus. Significant changes in 5 metabolites (GABA, glutamate, NAA, aspartate and scyllo-inositol) were observed in AD at two different ages (3–4 months and 8 months). Taurine moved a number of metabolites including NAA and glutamate closer to the wild-type profile consistent with neuroprotection. Overall, these findings support dietary taurine supplementation as a promising preventative strategy for AD.

## Introduction

Alzheimer’s Disease (AD) is a complex neurodegenerative disease resulting in memory loss, cognitive dysfunction and behavioral changes primarily in geriatric patients. Currently ~ 7 million Americans suffer from dementia and Alzheimer’s Disease (AD) with the number of cases progressively increasing^1,2^, placing what will be an unsustainable familial and economic burden upon society.

A number of FDA-approved medications exist to treat the progression of AD pathophysiology (i.e., preventing amyloid beta or tau aggregation) or its symptoms (cholinesterase inhibitors, glutamate regulators, antipsychotic medication)^3^. Unfortunately, due to the complexity of AD pathophysiology eliciting a multitude of biochemical and structural changes in the brain, often a single treatment against AD is not sufficient. There is a striking lack of success in modulation of chosen endpoints including both cognitive and imaging endpoints with high attrition rates. Between 2003 and 2021, only 2% of the AD drugs successfully advanced through phase II and III clinical trials^4^. This highlights the possibility that these trials are initiated too late, where symptoms emerge after the significant damage has already occurred^5^. As such, preventative therapies may be more effective than symptom mitigation or repairing extensive and complex pathology in late-stage AD. Numerous findings have demonstrated that both lifestyle factors such as education and exercise may be protective against AD, the latter being important due to the strong risk of AD associated with type II diabetes^6^. Further, a so-called Mediterranean diet,, is associated with lower risk of AD^7^. In addition, dietary or medicinal supplements such as non-steroidal anti-inflammatory drugs can provide protection against AD^8^. NSAID usage is associated, however, with negative gastrointestinal side effects^9^. Supplements that could be taken regularly, with high safety margins are thus attractive to investigate. Recent studies have observed that the endogenously produced amino acid, taurine, demonstrates neuroprotective potential. Taurine (2-aminoethanesulfonic acid), a cysteine-derivative and primarily responsible for osmoregulation, has been reported to reduce oxidative stress, inflammation, mitochondrial dysfunction and protect against glutamate excitotoxicity^10^, all characteristic of neurodegenerative diseases^11^. There is also evidence for protection in APPxPS1 AD mice using the taurine bile acid (TUDCA)^12^. Following glutamate, taurine is the second most abundant amino acid in the central nervous system, passing through the blood-brain-barrier via Na + and Cl− transport proteins^11,13^. Though most potential therapeutic effects of taurine have been observed in traumatic brain injury and stroke^14–17^, a few studies have also been conducted to elucidate the protective role of taurine in AD^18^. Recently, a study using glutamate PET observed that taurine may elicit therapeutic potential against AD through alteration of glutamate regulation in 5xFAD mice^19^. Similarly, taurine supplementation in the drinking water of APP/PS1 mice^20^ demonstrated positive effects on recovering spatial memory in late-stage AD. This work explores the protective role of taurine to slow down or prevent the progression of AD pathology by regulating both physiological and metabolic effects in 5xFAD mice.

Moreover, in addition to the hallmark AD pathophysiology such as Aβ and Tau aggregation, neuronal atrophy, and metabolic processes are also altered, classifying AD as a metabolic disorder^21^. Vital alterations in key metabolites (i.e., glucose, lipids, amino acids) can provide phenotypic insight into metabolic pathway disruptions contributing to the neuropathological hallmarks, such as plaque formation and neurofibrillary tangles, and AD symptoms^22^. Our lab and others have shown extensive loss of the neuronal marker NAA, as well as increases in molecules such as myo- and scyllo-inositol that are present primarily in glial cells in AD mice that mirror changes seen in human AD subjects^23–28^. By evaluating the changes in small molecule metabolism from homeostasis, biomarkers of AD can also be categorized and monitored in response to treatment procedures. This study uses MRS to classify metabolomic changes in key brain regions impacted by AD. Here, the metabolic changes following taurine treatment, in *ex vivo* brain tissue, are also explored to elucidate potential mechanisms of action of taurine protection. In addition, the use of MRS allows us to monitor changes in brain taurine levels.

The primary goal of this study is to determine if dietary supplementation with taurine can a) increase brain taurine levels, b) protect against Alzheimer’s disease (AD) pathology markers including markers of inflammation, hippocampal volumes and beta amyloid markers and c) protect against metabolic changes associated with the onset of AD pathology.

## Results and Discussion

### Effects of taurine supplementation on water consumption

**Figure 1:**
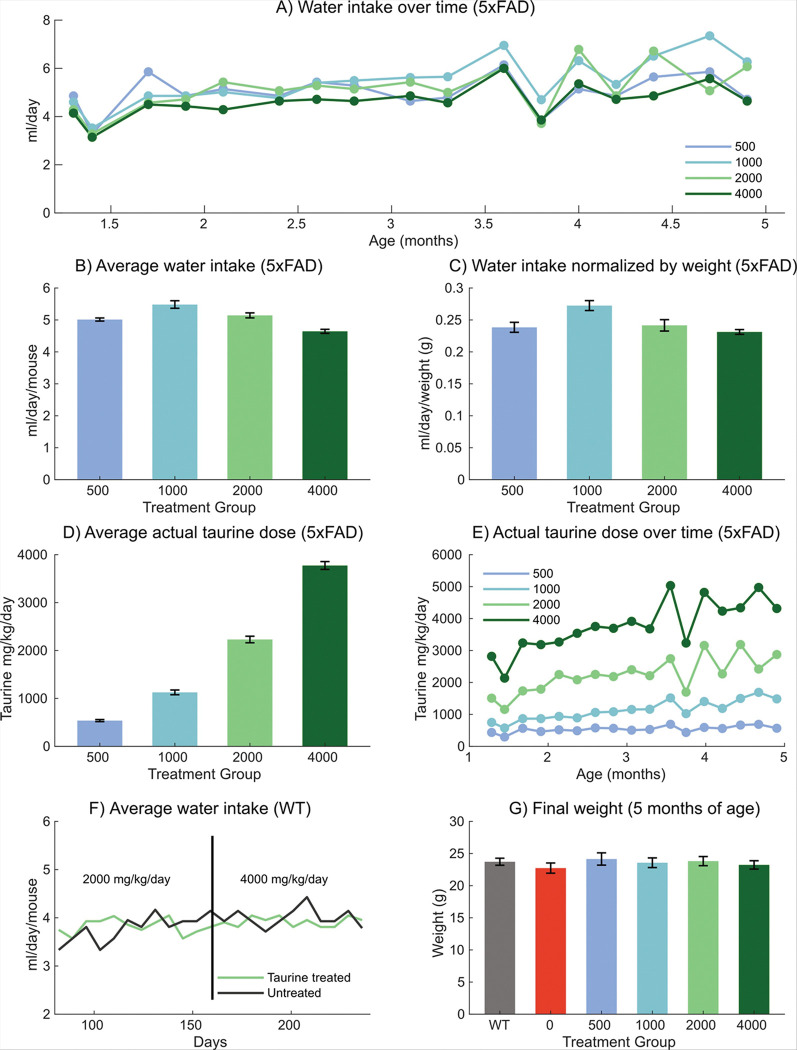
Water consumption of 5xFAD mice with taurine supplementation. **A)** Average water consumption (mL/day) using five concentrations of taurine (500, 1000, 2000, 4000 mg/kg, n=10 per group) over the course of 5 months. **B)** average water consumption per mice for each of the five taurine doses. **C)** Average water consumption per mice for each of the five taurine doses normalized to the animal’s weight. **D)** Actual taurine dose consumed by the animal calculated from the water consumed, over the course of 5 months **E)** Effect of two different doses of taurine on water consumption in WT animals (n=10 per group) **F)** Animals were treated with either control or treated water at 2000mg/kg/day and then switched to 4000 mg/kg/day (after the black line). There were no significant differences between the control and WT animals. **G)** Total body weight at 5 months of age for WT (n=10), untreated 5xFAD (n=9) and 5xFAD mice treated with varying doses of taurine (n=10 per group). There were no significant differences.

A key initial concern was whether taurine could be effectively delivered to 5xFAD mice via their drinking water. Figure 1 depicts the water consumption as a function of the taurine dose for both WT and 5xFAD mice. The average water consumption (mL/day) of each taurine dose in mice over 5 months was measured (Fig. 1A) by dividing the average water consumption per cage by the number of mice in each cage. Figure 1B and 1C show the average water consumption of each mouse with respect to the taurine dose, where Fig. 1C is normalized by the mean weight of the mice per cage. Here, the water consumption of the 1000 mg/kg dose was slightly higher than that of the 500 mg/kg (p < 0.05) and 4000 mg/kg (p < 0.002) doses.

The measured water consumption slightly varied between the actual (Fig. 1D) and intended doses, over 5 months (Fig. 1E). Here, the actual doses were calculated to be 538 mg/kg (500 mg/kg dose), 1118 mg/kg (1000 mg/kg dose), 2230 mg/kg (2000 mg/kg dose), and 3774 mg/kg (4000 mg/kg dose). The three lower doses (500, 1000 and 2000 mg/kg) were between 8–11% higher than the calculated dose while the highest dose (4000 mg/kg) was about 6% lower. This suggests that the water consumption must be monitored, and the taurine dose should be adjusted if necessary.

To confirm that taurine did not affect water intake, WT mice were monitored (Fig. 1F–G), while receiving a dose of 2000 mg/kg/day followed by 4000 mg/kg/day. An increase in the water intake was observed with the higher dose (4000 mg/kg/day), but this may be due to the increase in the animals’ weight as they age, thus requiring more water daily. Overall, taurine had no impact on water consumption or body weight over 5 months (Fig. 1G) in WT or 5xFAD mice, supporting its feasibility for supplementation via drinking water.

On the other hand, it was not clear, due to its postulated role in osmoregulation, whether water content would be stable after treatment. We therefore also measured the water content using HRMAS. Here the absolute value of water was the same in the taurine treated animals as the control (18.6 ± 3.0 vs. 16.7 ± 5.2 (arbitrary units); p = 0.36). As such, this avenue of taurine supplementation was chosen for the remainder of the experiments.

### Taurine dose determination via inflammatory cytokine markers

**Figure 2:**
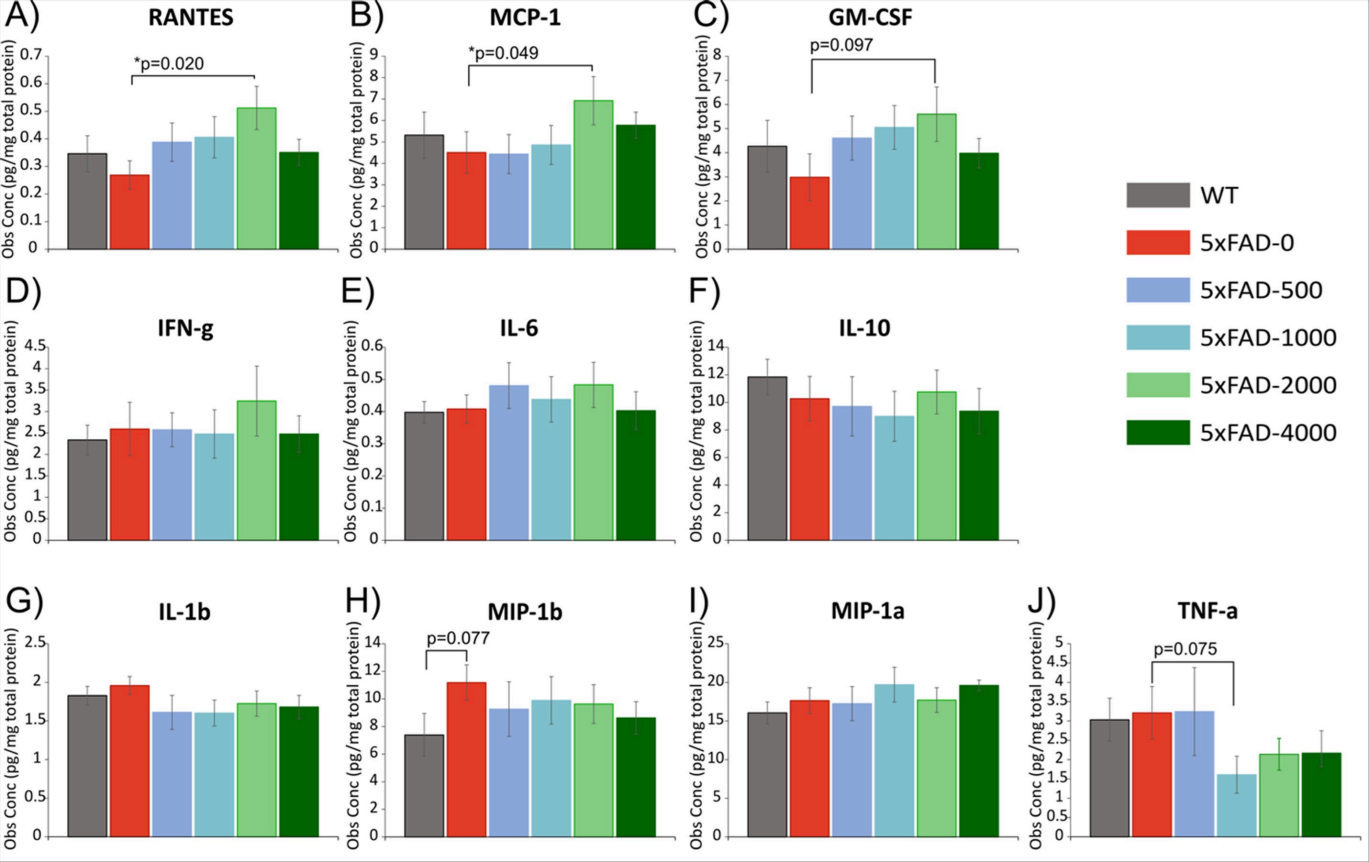
Effects of taurine treatment on cortical cytokine levels in 5xFAD mice. Luminex multiplex assay was used to analyze 10 cytokines/chemokines in the cerebral cortex of 5-month-old untreated transgenic 5xFAD mice (“5xFAD-0”, n=9), 5xFAD mice treated with taurine from 1–5 months of age at the doses of 500 mg/kg/day (“5xFAD-500”, n=10), 2000 mg/kg/day (“5xFAD-2000”, n=10), or 4000 mg/kg/day (“5xFAD-4000”, n=10), and 5-month-old non-transgenic wildtype mice (“WT”, n=10). Dunnett’s test was used to make multiple comparisons against the untreated 5xFAD control group. Data presented as mean pg/mg total protein ± SEM.

In the current project, we hypothesized that taurine treatment would reduce neuroinflammation in the 5xFAD mouse model of AD. Luminex multiplex assay (Millipore cat. MCYTOMAG-70K) was used to analyze 10 cytokines/chemokines in the cerebral cortex using protein lysates from 5-month-old untreated transgenic 5xFAD mice (n = 9), 5xFAD mice treated with taurine from 1–5 months of age at the doses of 500, 2000, or 4000 mg/kg/day (n’s = 10/group), and 5-month-old non-transgenic WT mice (n = 10) (Fig. 2). Dunnett’s test was used to make multiple comparisons against the untreated 5xFAD control group. There were no significant differences detected between untreated 5xFAD and WT mice on any of the cytokines measured at this age, which is in the early stage of the disease for the 5xFAD model. A pattern was detected in which 5xFAD mice treated with 2000 mg/kg/day showed an elevation in RANTES (p < 0.05), MCP-1 (p < 0.05), and GM-CSF (p < 0.10) compared to untreated 5xFAD mice (Fig. 2A–C). A similar pattern was also observed for IFN-g, IL-6, and IL-10 (Fig. 1D–F). There was also a significant effect of elevated RANTES from taurine treatment when all doses were analyzed in combination as compared to untreated 5xFAD mice (p < 0.05) (Supporting Figure S1). While RANTES is generally thought of as a pro-inflammatory chemokine, it is thought to have a neuroprotective role in AD, evidenced by an elevation in the cerebral microcirculation of AD patients and *in vitro* data showing that RANTES increases cell survival and protects against the toxicity from oxidative stress in neurons^29^. Thus, the taurine-induced elevation in RANTES in the 5xFAD mouse brain could be supportive of a neuroprotective response. MCP-1 is a chemokine expressed by microglia and macrophages, and plasma levels are associated with AD pathophysiology and cognitive decline in human patients^30^. Other evidence in AD animal models supports that MCP-1 deficiency impairs microglia accumulation around Aβ plaques and accelerates disease progression^31,32^. These studies support that early microglial accumulation in AD is dependent on MCP-1, possibly through the recruitment of macrophages from the blood and bone marrow to the brain. Thus, our data showing that the 2000 mg/kg/day dose of taurine leads to an elevation of MCP-1 in the early stage of the disease in the 5xFAD model could represent neuroprotective responses from the recruitment of plaque-associated microglia. Similarly, GM-CSF is typically thought of a pro-inflammatory cytokine, yet it has been shown to improve microglial function and Aβ clearance, and treatment with GM-CSF (drug name: sargramostim) results in improvements in AD patients in recent phase II clinical trials^33^. Thus, the elevation in GM-CSF observed from 2000 mg/kg/day taurine would be consistent with a positive treatment effect. IFN-g is also considered a pro-inflammatory cytokine; however, IFN-g levels are not reported to be increased in AD where in an AD mouse model, increased IFN-g expression leads to a suppression of Aβ levels in association with measures of improved glia function in phagocytosis^34^. IL-6 is a pleiotropic inflammatory cytokine produced by activated microglia and astrocytes, and in AD mouse models, overexpression is related to the attenuation rather than exacerbation of Aβ deposition^35^. IL-10 is considered an anti-inflammatory cytokine and important modulator of glia, preventing inflammation-mediated neurodegeneration, yet could be responsible for clinical worsening^36^. We detected a different pattern for IL-1b (p < 0.10) in which taurine-treated 5xFAD mice analyzed in combination (Supporting Figure S1) showed reduced levels (p = 0.052) compared to untreated 5xFAD mice (Fig. 2G). While there were no significant differences in MIP-1a (Fig. 2I) or TNF-a (Fig. 2J), a similar pattern was observed for TNF-a, with reduced levels at the higher doses of 2000 and 4000 mg/kg/day. IL-1b and TNF-a are both considered pro-inflammatory cytokines, and both are elevated in AD, specifically in response to tau^37^. Thus, the attenuation of IL-1b and TNF-a levels could be expected from a beneficial treatment effect. MIP-1a and MIP-1b are chemokines that have been weakly linked to AD. MIP-1b has recently been associated with cognitive decline in AD patients^38^, which could be consistent with our observation of decreasing trend for MIP-1b levels with taurine treatment (Supporting Figure S1).

Overall, our findings suggest that treatment of 5xFAD mice with taurine, particularly at the 2000 mg/kg/day dose, could be related to cytokine/chemokine functions involved in attenuating Aβ accumulation and preventing neurodegeneration in early stages of the disease. Using MRS, it was determined that taurine supplementation at this dose also increased hippocampal taurine levels (Supporting Figure S2). As the progression of Alzheimer’s disease likely hinges on the balance of the brain’s immune system between neuroprotective and neurotoxic effects – rather than simply increased inflammation – these changes in neuroinflammatory markers at later stages of the disease warrants further examination.

### Protective role of taurine on hippocampal microglia inflammatory response and structural integrity

**Figure 3:**
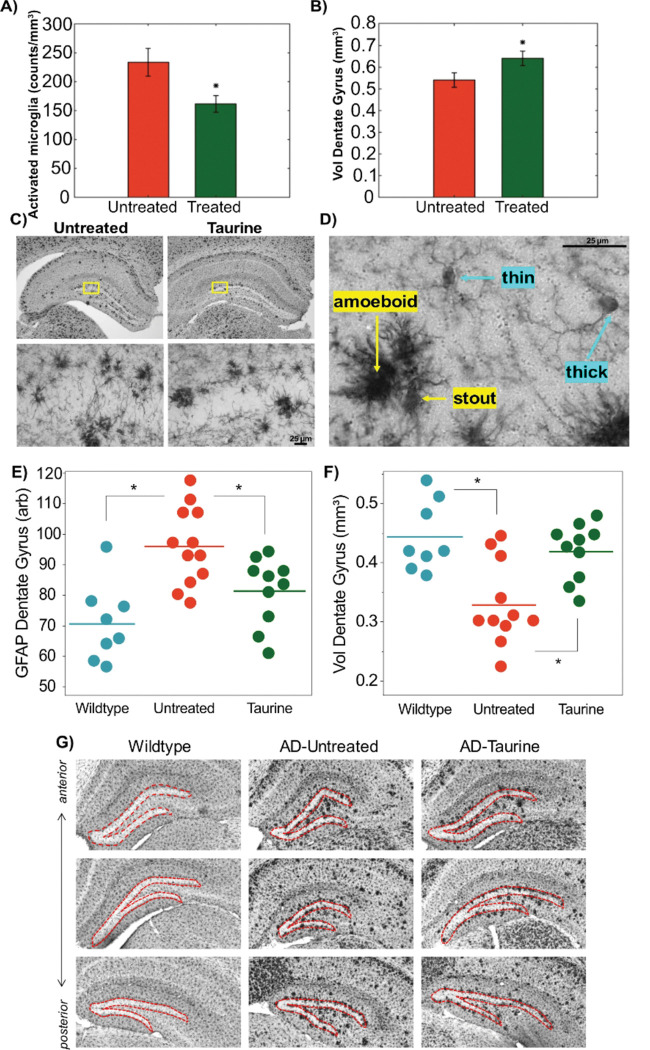
Protective role of taurine treatment in 5-month-old 5xFAD mice on reducing the number of activated microglia and the volume of the hippocampal dentate gyrus. **A)** Counts of Iba1+ cells with activated microglia morphology per volume of the dentate gyrus (DG) in 5xFAD mice that remained untreated (n=9) or were treated (n=10) with taurine at 2000 mg/kg/day from 1–5 months of age. *p<0.05 by Student’s T-test. **B)** The estimated volume of the dentate gyrus (DG) including the upper and lower blades, subgranular zone, and inner molecular layer across a series of three sequential sections (50 μm thick; 500 μm inter-section interval). *p<0.05 by Student’s T-test. **C)** Representative photomicrographs from untreated and taurine-treated 5xFAD mice and using a low magnification objective (4x, left) and a high magnification objective (40x, right). **D)** Photomicrograph using a high magnification objective (100x) capturing example microglia considered to have un-activated, ramified morphology (“thin” and “thick” labels) that were not counted and microglia identified to have activated or over-activated morphology (“stout” and “amoeboid” labels) that were counted for this analysis. Data shown as mean ± SEM. Scale bars indicate 25 μm. **E-G:** Protective role of taurine on 7–8-month-old 5xFAD mice hippocampal dentate gyrus GFAP **(E)**and volume **(F)**. G**)** The DG was outlined in sections containing both the dorsal and ventral blades of the DG. The DG area from each section (50 um thickness; 500 um intersection intervals). Photomicrographs from an Iba1 stain show the changes in DG area from anterior to posterior sections in three representative mice. Taurine was protective of the DG volume, as well as lowering GFAP staining.

Following our hypothesis that taurine treatment reduces neuroinflammation in 5xFAD mice and our cytokine results pointing to the most significant changes occurring at the 2000 mg/kg/day dose, we performed detailed analysis of microglial activation in the hippocampus between untreated 5xFAD mice and the 5xFAD mice treated from 1–5 months of age with the 2000 mg/kg/day taurine dose (Fig. 3). Microglia are considered the resident immune cells of the brain that undergo morphological changes and alter their production of cytokines in response to immune challenges, i.e., injury or disease. Under multiple challenges or increasing neuroinflammatory conditions, microglia transition from a “resting” state exhibited by thin, ramified processes that probe the environment to more “activated” states exhibited by a thicker morphology with shorter processes^39^. We focused on analysis of the hippocampal dentate gyrus (DG) because it is a well-defined subregion that is well-known for its function in memory formation and dysfunction that occurs in AD. Furthermore, evidence from AD mouse models^40,41^ and human post-mortem brain sections^42^ indicate that microglia activation in the DG is a disease-associated response to Aβ deposition. While initially the priming of disease-associated microglia is thought to have a neuroprotective role in containing Aβ plaques and clearing debris, over-activation leads to dysregulation that contributes to neurodegeneration^43^.

Using unbiased stereology, we found that taurine-treated compared to untreated 5xFAD mice exhibited a significant reduction (p < 0.05) in the number of DG microglia with an activated morphology (Fig. 3A). Additionally, we detected a significant elevation (p < 0.05) in the estimated volume of the DG subregion that was outlined for this analysis (Fig. 3B). For this analysis, the region-of-interest included the DG upper and lower blades, subgranular zone, and inner molecular layer surrounding the blades (Fig. 3C), and activated microglia were defined as those exhibiting a “stout” or “amoeboid” phenotype (Fig. 3D).

These findings indicate that taurine treatment beginning prior to disease onset and continuing into the early disease stage of the 5xFAD model mitigates neuroinflammation as measured by the characteristic microglial activation that occurs in response to Aβ deposition. Taken together with the cytokine/chemokine changes at this dose of 2000 mg/kg/day, taurine could be involved in improving the neuroprotective functions of microglia, preventing or delaying a transition into the dysregulated neuroimmune signaling that is associated with AD.

Thus far, we have discussed administrating taurine long-term as a preventative measure; however, this may not be feasible for geriatric patients where the pathology has already progressed prior to the onset of symptoms. Here we explore the protective role of acute taurine administration on hallmark AD pathological characteristics, where the animals were only supplemented with taurine for 1–2 months. Figure 3E–G shows the protective effects of taurine treatment in 7–8-month-old mice treated with 2000 mg/kg of taurine from the age of six months. GFAP, a glial marker upregulated in AD-affected brain regions^44,45^, was used to assess taurine’s effects. While it supports astrocyte-neuron interactions^46,47^, elevated GFAP levels can indicate astrocyte dysfunction, often associated with neurodegeneration and cognitive decline. In this study, there was also a trend of taurine treatment to protect GFAP in the 3–4 month old animals in the hippocampus (GFAP % Area of Control: 10.8 ± 0.7 vs AD: 14.9 ± 1.7 vs AD-Taurine: 13.98 ± 1.6, p_AD vs AD−Taurine_ = 0.88 using a post-hoc Tukey HSD) but it did not reach significance given that the GFAP upregulation in those mice is substantially smaller than that observed in the 7–8 month old animals^45^. Relative to the WT, the untreated 5xFAD mice observed a 36% increase (p < 0.001) in microglia activation (Fig. 3E), where taurine treatment demonstrated a protective role in the dentate gyrus GFAP, with a 19% reduction (p < 0.05) on activation relative to the untreated 5xFAD mice.

Moreover, the reduction in DG volume may precede the formation of amyloid beta plaques characteristic of AD^48^. Here, the volume of the dentate gyrus in untreated 5xFAD mice (n = 11) reduced by 38% (p < 0.001) relative to the WT mice (n = 8) as seen in Fig. 3F. This is visually reflected in the IBA-1 images in Fig. 3G. Interestingly, following taurine supplementation (n = 10), the dentate gyrus volume returned (p < 0.01) to near normal levels (WT: 0.44 mm^3^ vs taurine-treated 5xFAD mice: 0.42 mm^3^). This showcases the advantages of taurine in preserving the structural composition of key brain components and thereby AD pathophysiology. Overall, this study demonstrates that short-term taurine supplementation (1–2 months) protects against glial activation and preserves dentate gyrus volume, supporting structures essential for learning and memory.

### Protective role of taurine on amyloid beta pathology

**Figure 4:**
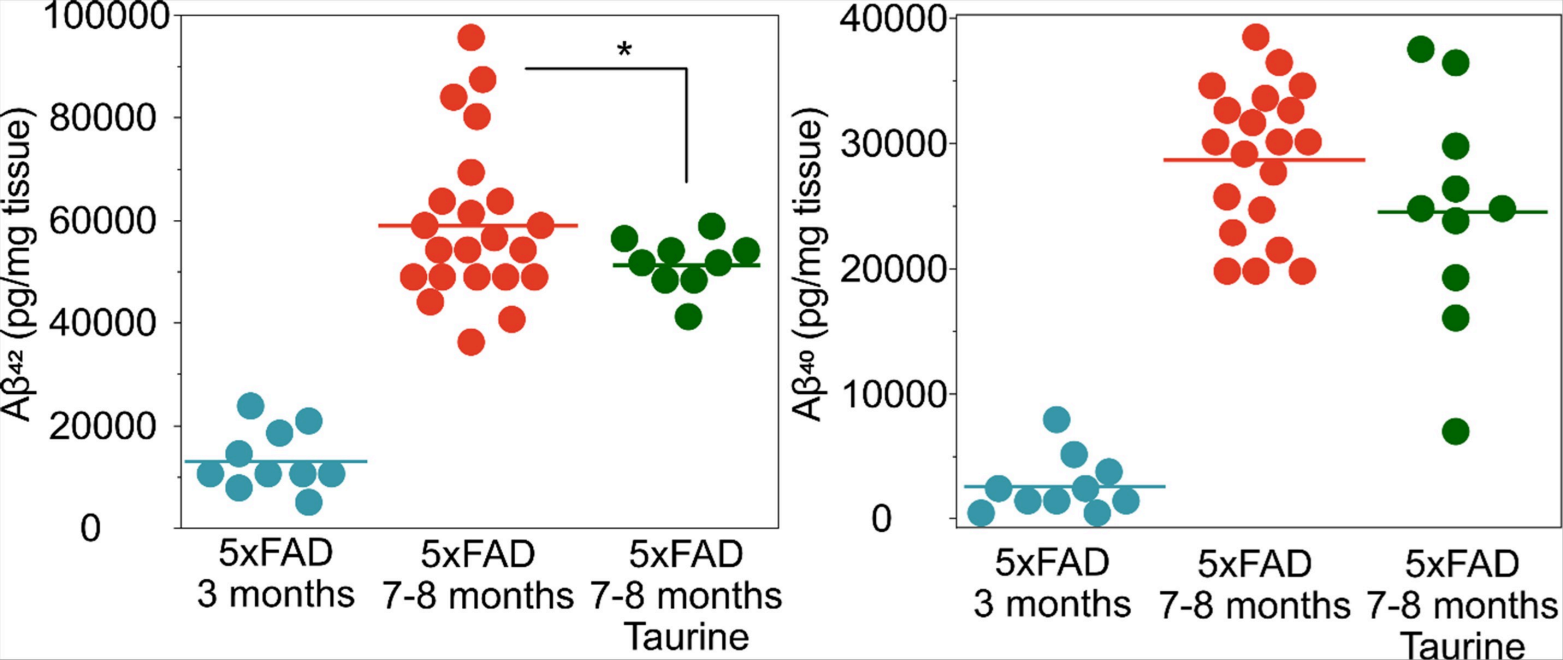
Plots of ELISA measures of amyloid beta (42 and 40) from hippocampus in the 5xFAD mice at 3 months and 7–8 months of age (for Aβ42: 3 months old untreated 5xFAD n=10, 7–8 months old untreated 5xFAD n=22, 7–8 months old treated 5xFAD n=9. For Aβ40: 3 months old untreated 5xFAD n=10, 7–8 months old untreated 5xFAD n=20, 7–8 months old treated 5xFAD n=10). The treated mice were treated with 2000 mg/kg starting at 6 months of age. There was a statistically significant decrease in Aβ42 (p=0.04). There was a trend towards lowering Aβ40 – with some of the mice being quite low, but because of the high variance it did not reach statistical significance.

It is well known that amyloid beta accumulation is a hallmark of AD. As the 5xFAD mice are engineered to develop amyloid pathology, in addition to the changes in other markers of neuronal metabolism and function, we investigated the effects of taurine treatment on amyloid deposition in the hippocampus. As expected, there is a large increase in amyloid deposition (Aβ42 and Aβ40) in the brain over time with a large change between 3–8 months of age as shown in Fig. 4. Acute treatment with taurine until 7–8 months (starting from six months of age) resulted in a small decrease in Aβ42 (Fig. 4A, p = 0.04) levels. Aβ42 is considered to be the more toxic of the two amyloid fragments^49^, with increased propensity for aggregation and neurotoxicity. Likewise, there was also a trend towards a decrease in Aβ40 levels (Fig. 4B), however, due to the small sample size effect and high variance this did not reach significance. A study in cell cultures observed the protective role of taurine against neuronal loss due to Aβ42 and recovered mitochondrial dysfunction^50^. Interestingly, studies have also noted no change in amyloid beta burden in the hippocampus with taurine treatment^19,20^, but rather the interaction between taurine and oligomeric Aβ aggregation driving the recovery of cognitive deficits^51^. Although these results are encouraging, based upon the relatively small effect size it can be assumed that neuroprotection with taurine supplementation is not primarily working through an amyloid beta mechanism.

### Determining brain regions of interest for metabolomic studies

The cytokine and glial inflammatory responses alongside changes to amyloid plaque behavior have provided insight into the protective role of taurine in the broader context of AD pathobiology. However, metabolomic information can further improve our understanding of the mechanism of protection at the biochemical level. The hippocampus and cortex have long been associated with symptoms of AD; however, these regions of the brain are large, encompassing many sub-regions with distinct metabolomic profiles. Here, we observe changes in the metabolic profile associated with 3 specific hippocampal and cortical regions relevant to AD in both 5xFAD and WT mice; the frontal cortex (FrCx), dorsal hippocampus (DHP) and ventral hippocampus (VHP). Here, mice between the ages of 3–4 months were used to represent ‘early-stage’ AD pathophysiology. This may also provide vital information regarding early-onset AD where biochemical changes can precede symptoms at early stages. All three of these regions are strongly affected by plaques in the 5xFAD mice as the pathology progresses. It is valuable to see what differences in the baseline exist between these brain regions to determine what one might expect to see with AD pathology and taurine protection.

**Figure 5:**
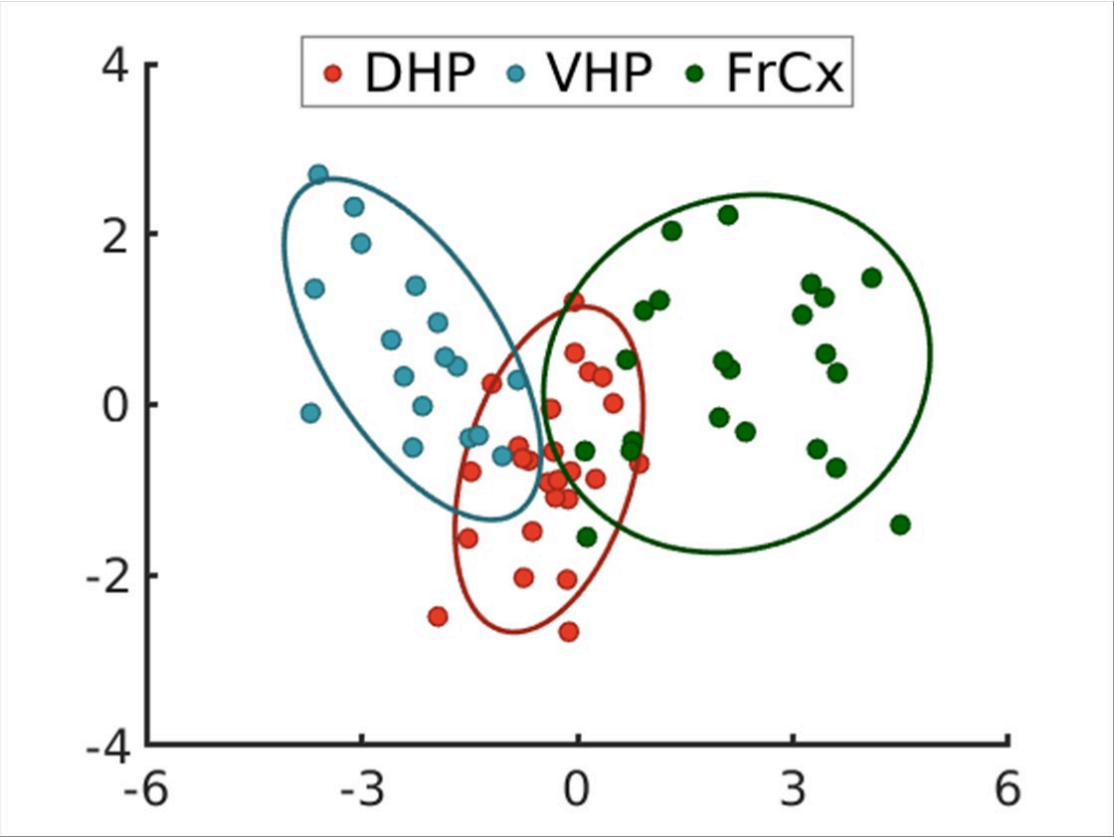
Separation of three brain regions by metabolic profiles in 3–4-month-old WT mice; Red - dorsal hippocampus (DHP), Blue - Ventral Hippocampus (VHP), Green - Frontal cortex (FrCx). Linear discriminant analysis using five neurochemical feature variables (aspartate, glutamate, N-acetyl-aspartate, scyllo-inositol and myo-inositol) showing that the DHP is closer to cortex than VHP. The ellipses represent 95% confidence bounds. Separations in axis 1 (x axis) were significant but not in axis 2 (y axis). Also shown are the confusion matrices for a multi-layer perceptron and support vector machines classifiers using hold out analyses. All classifiers put the VHP further from the FrCx than either is to DHP.

In general, there are some major differences between the brain regions in terms of the distribution of neurochemicals, as can be seen in Fig. 5. Machine learning was used to differentiate the metabolic profiles of the three brain regions. Using a feature variable selection process (Relief-f), the total list (16 metabolites) of metabolites was narrowed down to the five chemicals (aspartate, N-acetylaspartate (NAA), glutamate, GABA, and scyllo-inositol) to be used as classifiers. A multi-layer perceptron, linear discriminant (LDA) and support vector machines classifiers with the five chemicals was used with at least 17 data points per group and a jackknifing analysis was performed. The multi-layer perceptron correctly classified 83% of the data points relative to support vector machines with 80% correct classification. Figure 5 depicts a plot of the LDA classifier in the WT mice showing that the largest distance between groups is between VHP (n = 17) and FrCx (n = 22), where the metabolic profile of the DHP (n = 25) was found to closely resemble the FrCx rather. Due to the distinct profiles between the FrCx and VHP, these two regions were chosen for further MRS study at baseline and with taurine treatment.

The metabolic changes attributed to AD were also determined for both FrCx and VHP. Table 1 summarizes the neurochemicals distinguishing between the FrCx and VHP and depicts their trend arising from AD pathology. This information can be used to understand the impact of taurine treatment on region-specific neurochemicals.

Interestingly it was found that NAA, a marker of neuronal health and viability, was significantly higher in the cortex (NAA/Cr = 0.89) than either the DHP (NAA/Cr = 0.66) or VHP (NAA/Cr = 0.53). This suggests that during AD, NAA may potentially decrease to a greater extent in the FrCx than in the VHP. This is supported by the findings in Table 1, where during early AD, NAA decreased by 18% in the cortex (p = 0.03) compared to the VHP where NAA decreased by 11% (p = 0.05). The lower NAA levels in the hippocampus compared to the FrCx may be reflective of the fact that, although some sub-regions of the hippocampus have larger neuronal densities than the FrCx, the overall hippocampus has a lower neuronal density^52^. The larger decrease in NAA in the FrCx relative to the VHP may be reflective of prior studies where cortical thinning rates accelerate during early/presymptomatic AD stages whereas hippocampal atrophy progressively accelerates over the course of the disease^53^.

Glutamate and aspartate levels were higher in the FrCx than in the VHP, though in the 5xFAD mice their levels only decreased in the cortex (glutamate − 13%, aspartate − 14%) and remained unchanged in the VHP. Both metabolites are excitatory neurotransmitters predominantly found in neurons^54,55^. As aspartate and glutamate levels are higher in neurons relative to astrocytes, their decrease in 5xFAD mice may reflect neuronal loss, a hallmark of AD pathology^54,55^.

Likewise, GABA, an inhibitory neurotransmitter, was found in similar levels in both FrCx and VHP, where it was seen to significantly decrease in both the FrCx (−15%) and the VHP (−10%) during AD. Similarly, GABA is more abundant in neurons relative to astrocytes^49^, and this decrease is reflective of neuronal loss, with cortical thinning occurring faster than hippocampal atrophy^47^. These changes in neurotransmission may also contribute to the behavioral deficits of AD.

On the other hand, scyllo-inositol is present in both FrCx and VHP though concentrations are higher in hippocampus as we published previously^1^ but was seen to increase by similar amounts in both the cortex (+ 51%) and VHP (+ 50%). This is consistent with prior studies in mice showing higher microglial density in hippocampus than FrCx^56^ as myo- and scyllo-inositol are not found in neurons.

### Protective role of taurine on neurochemical profiles

**Figure 6:**
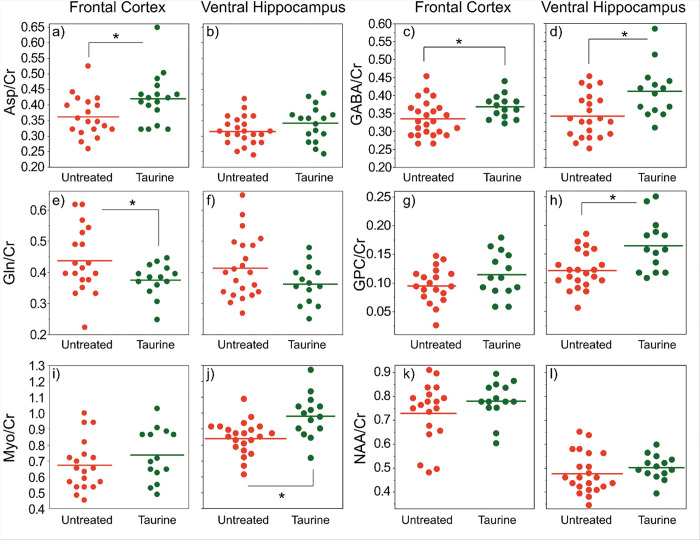
Plots of alterations in neurochemical profiles within FrCx and VHP in 3–4 months old untreated 5xFAD mice (n= 19 for FrCx, n=22 for VHP) vs. treated (n=14) with 2000 mg/kg taurine from 1 month of age 5xFAD mice. Shown are the chemicals that had significant changes in at least one brain region (except for NAA the neuronal marker). Chemicals on the left of plots.

By establishing the metabolic profile of both WT and AD mice (3–4 months old), the effects of taurine supplementation (2000 mg/kg/day) in the two brain regions (VHP and FrCx) were compared. Here ex vivo analysis via HRMAS spectroscopy was used to understand the biochemical changes following taurine treatment (starting from 1 month old). These findings can help provide complementary information for future *in vivo* work. At a dose of 2000 mg/kg/day, taurine displayed a protective response on a number of important neurochemicals in both FrCx and VHP, depicted in Fig. 6.

**Figure 7:**
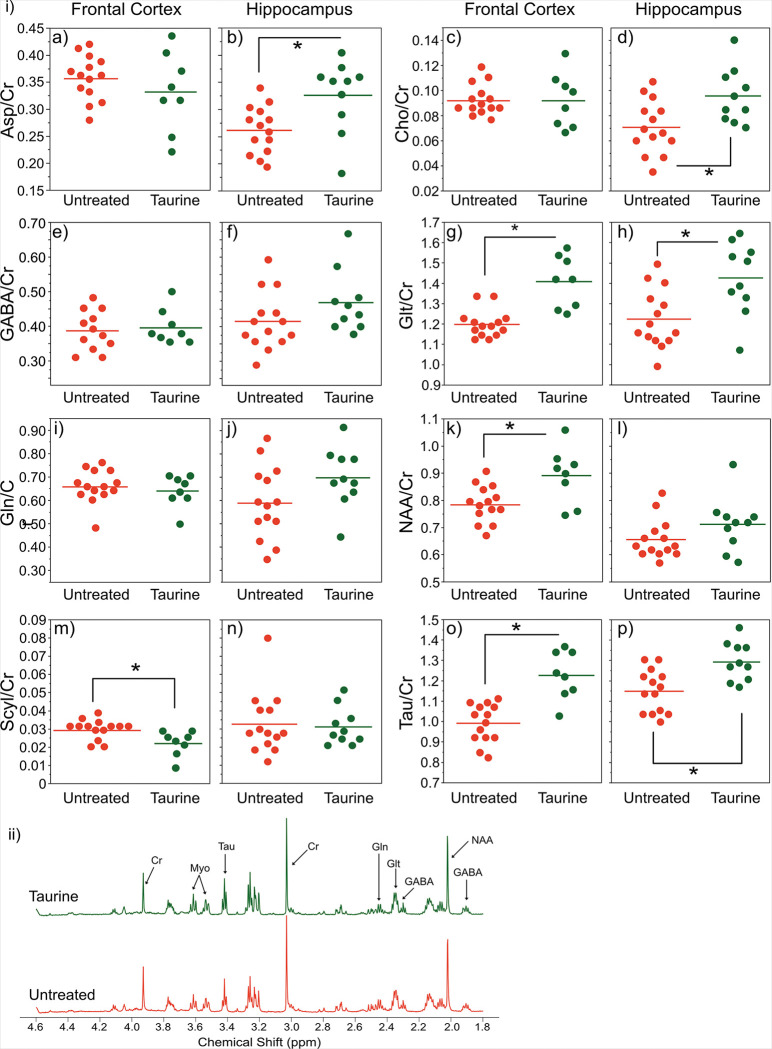
Effects of taurine (tau) supplementation in cortex at 8 months of age. i) Plots of metabolites in the frontal cortex and hippocampus with (Cortex=8, Hipp =10) and without (Cortex=14, Hipp=14) taurine supplementation. ii) Averaged HRMAS spectra from tau-treated (blue) and regular diet 5xFAD mice (red). Taurine can be seen to increase following supplementation.

The general pattern of alterations in the taurine treated animals was the same in both FrCx as well as VHP; however, the magnitude of changes varied by metabolite. Figure 6 only shows the neurochemicals for which there was a significant change with taurine treatment, except for NAA where there was no significant change at this age but was at 8-months (Fig. 7). This suggests that taurine’s protective role on neuronal health could potentially be by mitigating NAA loss. A light scattering and electron microscopy study found that NAA was able to break up fibrils of aggregated amyloid beta^57^.

The decrease in GABA noted in the 5xFAD mice (Table 1) is also protected by taurine treatment in both the FrCx and VHP (Fig. 6c–d). Aspartate, which decreased significantly in the FrCx of the untreated 5xFAD animals, is increased with taurine treatment in both brain regions (Fig. 6a–b). This increase in aspartate and GABA may also correlate to the protection of neurons in both brain regions. Increased aspartate levels can also stimulate the production of glutamate and glutamine in astrocytes^58^. Glutamine, which is unchanged (Fig. 6e–f) in the untreated 5xFAD animals at this age (but increases later, Fig. 7), is decreased by taurine treatment. This change may reflect the conversion of glutamine to GABA after it is shuttled out from astrocytes into neurons^59^.

Moreover, myo-inositol, a compound increased in human AD brains, was seen to increase following taurine treatment (Fig. 6i–j). The increase in myo-inositol and in scyllo-inositol is thought to confer some protective properties for the cells, by inducing the formation of stable Aβ42 micelles, preventing fibril formation^1,2^. Alternatively, as myo-inositol is produced specifically in glial cells (not in neurons), an increase in its level may be related to gliosis, characteristic of AD. This suggests that taurine may not be effective at reducing gliosis in early AD but serves to protect gliosis in later stages (Fig. 4). Whether this increase at 3–4 months is a result of AD pathology or a protective response requires further investigation.

In this study, an increase in glycerophosphocholine (GPC, Fig. 6g–h) with taurine treatment was detected in both brain regions. Elevated GPC levels have been reported in CSF^60^ alongside postmortem tissue^61,62^ of AD patients, reflecting the relation between neural lipid membrane disruption and AD. Though its levels were increased following taurine treatment, GPC is a precursor to acetylcholine^63^, a neurotransmitter responsible for learning, memory and cognitive function, and this increase may be a protective response to AD pathology. Additionally, GPC intake demonstrates potential protective roles on the blood brain barrier function and decreasing microglial activation in SAMP8 mice^64^. This suggests that taurine treatment may influence GPC levels to increase cholinergic neurotransmission or improve the stability of neural membranes.

### Protective role of taurine in late-stage AD

Thus far, the results have shown that consistent taurine supplementation from an early age demonstrates several protective functions. We were curious if the same protection can be achieved through short-term administration of taurine in prolonged/late-stage AD pathology at older ages (Fig. 7). Here, 6-mo 5xFAD mice were supplemented with taurine for 2 months, and HRMAS MRS was used to observe the metabolic profile of tissue from both the frontal cortex and hippocampus at 8-months of age (reflective of late-stage AD in humans).

We observed significantly (p < 0.05) increased levels of NAA in the cortex (Fig. 7i–k), as well as taurine in both cortex and hippocampus (Fig. 7i– o & p, and Fig. 7–ii) of the 8-month-old 5xFAD mice. Thus, even short-term supplementation of taurine initiates neuroprotective responses in a progressed disease state.

Moreover, we observed an increase in aspartate in the hippocampus (Fig. 8i-b) and glutamate in both cortex and hippocampus (Fig. 7i– g & h), reflective of taurine’s neuroprotective response as both metabolites are predominately found in neurons (relative astrocytes). Prior studies showed a decrease in the metabotropic glutamate receptor in both human^65^ and mouse^19^. Interestingly, we noted a very strong correlation between the taurine levels and glutamate levels in both frontal cortex and hippocampus (Fig. S3). The increase in aspartate can also promote the production of glutamate and glutamine in astrocytes^58^ and may also relate to N-methyl-D-aspartate (NMDA) receptor function and therefore memory formation^66^.

Moreover, scyllo-inositol levels were also increased in the untreated 5xFAD animals relative to wildtype (p < 0.05, also seen in the 3–4 months old animals, Table 1), and this may suggest a protective compensatory response as mentioned earlier. The reduced scyllo-inositol levels in the frontal cortex of taurine treated mice (Fig. 7i–m) might suggest this mechanism of protection was not initiated as taurine helps prevent AD pathology.

Something interesting to note; upon comparing metabolite concentrations in the frontal cortex, we noticed that some of the untreated 5xFAD animals demonstrated reduced creatine and glutamate levels (relative to wildtype animals, p < 0.05) in the several tissue samples – due to such low creatine values, we deleted these mice from analysis. This was only observed in the 8-month-old animals and not at the younger ages (3–4 months).

Nevertheless, in all cases, taurine treatment moved the animals’ metabolite levels closer to the wildtype values. Overall, our findings support that taurine supplementation does play a protective role in metabolites related to neuronal health.

## Conclusion

Overall, this study explored the protective effects of taurine in 5xAD mice, showing that supplementation via drinking water had no impact on consumption, body weight or tissue water content. Alterations in the inflammatory cytokine profile and the number of activated microglia demonstrated that a 2000 mg/kg/day dose was sufficient to elicit a protective response in 5-month-old 5xFAD mice. This dose was also observed to protect against the reduction of the dentate gyrus volume as well as gliosis through the production of GFAP in 8-month-old mice. Taurine was also found to reduce Aβ42, albeit with a small effect size, in 7–8 months-old mice. Moreover, machine learning was used to determine which brain region to target for further studies, where the frontal cortex, ventral and dorsal hippocampus were found to elicit changes in 5 different AD-related metabolites (GABA, glutamate, NAA, aspartate and scyllo-inositol). The frontal cortex and ventral hippocampus were determined to be the most different from each other, and HRMAS was used to assess taurine’s protective effects on AD-related metabolite changes. In general, taurine exerts a protective response against neuronal loss. Future work will utilize both MRI and MRS to correlate both structural and metabolic changes in 5xFAD mice, while elucidating the protective response of taurine in these brain regions longitudinally. Taurine supplementation holds the potential to be a viable treatment option to protect against biochemical alterations due to AD pathophysiology.

## Methods and Materials

### Animals

All work described in this manuscript has been performed in compliance with the Mass General Brigham (MGB) and the Institutional Animal Care and Use Committee (IACUC) ethical guidelines for animal handling and experimentation. In addition, the work in this study is reported in accordance with ARRIVE guidelines (https://arriveguidelines.org).

Female 5xFAD transgenic mice and non-transgenic female littermates as controls were used in this study. These mice were bred from our mouse colony that originated with 5xFAD mice [B6SJL-Tg(APPSwFlLon,PSEN1*M146L* L286V)6799Vas/Mmjax] purchased from the Jackson Laboratory (Bar Harbor, ME), an NIH funded strain repository, and was donated to the MMRRC by Robert Vassar, Ph.D., Northwestern University^67^. 5xFAD mice express human APP and PS1 genes, harboring a total of five familial AD mutations [APP K670N/M671L (Swedish) + I716V (Florida) + V717I (London) and PS1 M146L + L286V] under the control of the murine Thy1 promoter. Mice were grouped-housed (4–5 per cage) in ventilated shoebox cages and kept on a 12 h light:12 h dark schedule. All mice were given access to standard rodent chow and drinking water ad libitum. All animal experiments were performed in accordance with the NIH Guide for the Care and Use of Laboratory Animals and were approved by the research animal care committee at the VA Boston Healthcare System and Mass General Brigham.

### Treatment protocol

All animals were randomized to the taurine/no taurine treatments. Taurine (cat. T8691) was purchased from Sigma-Aldrich (St. Louis, MO). Taurine was dissolved in the drinking water at four different doses (500, 1000, 2000, and 4000 mg/kg/day) assuming an average consumption of 5 ml/day per mouse. Water bottles were changed weekly. Body weights and water consumption were monitored weekly. Mice were monitored daily for general well-being, and no side-effects related to taurine treatment were observed. A total of 60 animals were used; however, 1 animal died prior to analysis (total = 59 animals). Animal numbers were chosen based upon a minimal sample size All animals were kept in the same location, (4 animals per cage), and were treated on the same day to minimize confounders. Animal numbers per group are given in the text and figure legends. Due to the number of studies we have performed in these mice we can performed a power analysis for the minimum numbers of mice needed to perform the studies The numbers seen outlined in the various experimental sections were determined from power analyses using the G* package (freely available from the Heinrich Heine University of Dusseldorf) for the proposed experiments. We calculated the numbers of animals using a simple one-way ANOVA with corrections for multiple comparisons. With 10 animals per group we would have (for an ANOVA fixed effects, main effects and interactions) with a power of 0.8 to detect an alpha of 0.05 we could measure an effect size of 0.28 (measured as Cohen’s f). We have gone over 10 animals per group in some cases to lower the overall effect sizes we could obtain within the limits of what we can predict.

### Tissue collection

Euthanasia was performed by CO_2_ asphyxiation consistent with guidelines for euthanasia of rodents using CO_2_

https://oacu.oir.nih.gov/system/files/media/file/2024-01/b5_euthanasia_of_rodents_using_carbon_dioxide.pdf).

The left hemisphere of the brain was dissected, flash-frozen on dry ice, and stored at −80°C for the analysis of the following: the prefrontal cortex (PFC, from bregma levels 3 to 1) to prepare protein extracts for the analysis of Aβ by enzyme-linked immunosorbent assay (ELISA), frontal cortex (FC, circa bregma 1 to 0) for high resolution magic angle spinning spectroscopy (HRMAS), the remaining cortical levels (CTX, bregma levels 0 to − 4) for multiplex cytokine ELISA analysis, and at the ventral hippocampus/subiculum (VHP, circa bregma − 3.5) for HRMAS. The right hemisphere of the brain was post-fixed with 4% paraformaldehyde solution for 24 h, cryoprotected in a graded series of 10% and 20% glycerol in a 2% DMSO solution and stored at 4°C prior to histological processing.

### Multiplex cytokine ELISA

Cortical tissue (CTX) was homogenized with 3 volumes (w/v) of complete lysis M buffer (cat. 04719956001, Roche) and centrifuged at 20k × g for 15 min to extract the supernatant. Total protein levels were measured using a BSA protein assay kit (cat. 5000002, Bio-Rad). Samples were analyzed on a multiplex panel of inflammatory cytokines and chemokines (RANTES, MCP-1, GM-CSF, IFN-γ, IL-6, IL-10, IL-1β, MIP-1β, MIP-1α, and TNF-α) using the Milliplex mouse cytokine immunoassay according to the manufacturer’s specifications (cat. MCYTOMAG-70K, MilliporeSigma, Burlington, MA) and as previously described^40^. The results were read on a Luminex 200 (Luminex, Austin, TX) using xPONENT software version 4.3 (Luminex) and analyzed using Belysa analysis software version 1.1 (Merck, Darmstadt, Germany). Briefly, samples were added into the wells of a pre-washed 96-well plate at 40 μg protein/well. Assay buffer and magnetic beads were then added to each well. After an overnight incubation at 4°C, the plate was washed with a handheld magnet and incubated with the detection antibodies for 1 h at 25°C. Wells were then developed by the addition of a streptavidin-phycoerythrin fluorescent labeling agent. The plate was then washed and read on the Luminex 200 to obtain the median fluorescent intensity (MFI) data.

### Histology and immunohistochemistry (IHC)

The right hemispheres were serially cut at 50 μm thickness on a freezing microtome. Serial sections 500 μm apart were immunostained as previously described^68^. In brief, free-floating sections were incubated overnight in primary antibody followed by phosphate-buffered saline (PBS) washes and incubation in peroxidase-conjugated secondary antibody, followed by development using 3,3′-diaminobenzidine tetrahydrochloride (DAB) as a chromogen. The antibodies used were against ionized calcium binding adaptor molecule 1 (Iba1) (1:5000, rabbit polyclonal, cat. 019–19741, Wako Chemicals, Richmond, VA) to stain microglia and glial fibrillary acidic protein (GFAP) (1:5000, mouse monoclonal, cat. MAB3402, Chemicon, Temecula, CA) to stain reactive astrocytes.

As microglia become activated during an inflammatory response, these cells undergo morphological changes indicating increased activation states, which can be quantified using unbiased stereology as a measure of microglial activation. To estimate the number microglia that were activated in the Iba1-stained sections, we used the Optical Fractionator probe in StereoInvestigator software (MBF Bioscience, Williston, VT) and outlined the DG region-of-interest (ROI) including the upper and lower blades, subgranular zone, and inner molecular layer using the 4x objective. For identifying activated microglia, we defined activated microglia as those with a “stout” or “amoeboid” morphology, exhibiting an enlarged, rounded cell soma with short, bushy processes. The mean estimated density of activated microglia (counts/area) and estimated DG volume was obtained from three anterior hippocampal sections (500 μm intersection interval) of each individual observed using the 40x objective.

Astrocytes become reactive in an inflammatory response and overexpress GFAP, such that the expression level of this protein, which can be analyzed using densitometry as a measure of astrocytic reactivity. GFAP densitometric analysis was performed using photomicrographs of GFAP-stained sections captured using the 10x objective containing the DG ROI (500 μm intersection interval) using ImageJ software (NIH, Bethesda, MD). On ImageJ, the ROI was outlined and a standard threshold value was applied to capture the majority of the GFAP immunoreactivity across all individuals. An analysis of all particles captured with the threshold produced the GFAP densitometry measures (%area stained) and the volume of the DG was estimated using the measured ROI areas from three sections per individual.

### Aβ enzyme-linked immunosorbent assay (ELISA)

Dissected prefrontal cortex (PFC) brain tissue was homogenized with 10 volumes (w/v) of cold 5 M guanidine HCl buffer. Specific human Aβ40 and Aβ42 ELISA kits (Invitrogen cat# KHB3544 and KHB3545) were used according to the manufacturer’s specifications. Briefly, homogenized samples were added into the wells of a pre-treated 96-well plate. Samples were then mixed with a cleavage-specific antibody to either Aβ40 or Aβ42. After an overnight incubation at 4°C, plates were washed and incubated with the secondary antibody for 30 min at 25°C. Washed wells were developed by the addition of a substrate. The substrate reaction was then stopped, and color intensity was measured at 450 nm.

### High resolution magic angle spinning spectroscopy

As previously published^69^, proton magnetic resonance spectroscopy (^1^H-MRS) was collected using high resolution magic angle spinning (HRMAS) spectra on Bruker 14 T (Billerica, MA). Viable samples were obtained from dissected tissue punches from the frontal cortex (FrCx) and ventral hippocampus/subiculum (VHP) of the mice. Samples were placed into a glass cylinder positioned in a 3 mm zirconium oxide MAS rotor (volume: 50 μL). HRMAS measurements were performed using a sample spinning rate of 3.6 kHz selected to push the spinning side bands outside the frequency region of the metabolites. The experiments were performed at 4°C to minimize tissue degradation. Data were acquired using a rotor synchronized, T2-filtered Carr–Purcell–Meiboom–Gill (CPMG) pulse sequence [90 − (τ − 180 − τ − Acq)n] with two different effective TEs (100 ms/10 ms). The longer TE serves to remove the lipid/macromolecular resonances, and the short TE retains them. The interpulse delay, τ, is synchronized to the rotor frequency, and is 272 μs. The n value for the relatively short T2 filter was 36 and for the long TE was 360. The short τ value removes all the T2* - like effects on the line shapes. The long T2 filter yields approximately 95% of the total spectral intensity of all metabolites of interest compared to the short TE. Other acquisition parameters were a 90° pulse of 5–10 μs, a spectral width of 8 kHz, 16 K complex points, 256 averages and a TR of 5 s. Samples were placed in the rotor with a small amount of D_2_O (Sigma-Aldrich, Milwaukee, WI) for locking and shimming. All animal MRS data was blinded and coded by one investigator (ZMS) such that the person doing the data analysis (BGJ) did not know the treatment status. Data were analyzed using the Chenomx (Edmonton, Alberta, Canada) package fitting the entire metabolite spectrum for each neurochemical and reported as molar ratios to creatine. Classification of the data was performed using the WEKA package^70^.

### In vivo MRS

WT animals (total n = 8) were treated with taurine (n = 4) and compared to those without taurine supplementation (n = 4). Animals were anesthetized using 1–1.4% isoflurane for the duration of the experiments. In vivo MRS (PRESS at 9.4T, repetition time (TR) 2.2s with an echo time (TE) of 16ms) with a single voxel in the hippocampus including the dentate gyrus, CA1 and CA2, that was approximately 2 × 1.5 × 1.3 mm (39 μL) was used, consisting of 500 averages. Data shown in Supplementary material.

### Machine learning for discrimination between treatment groups

We utilized a Relief f algorithm and correlation feature selection to identify which feature variables contributed the most to the separation between the treatment groups so that we could reduce the number of variables to a level appropriate for the numbers of animals. These variables were then used in well-established machine learning classification techniques to discriminate between groups including linear discriminant analysis (LDA), support vector machines (SVM) or multi-layer perceptrons (MP), or k-means clustering all performed with hold-out analysis using the 3–4 highest ranked attributes to gain additional information about classification between groups. The data were analyzed using the WEKA package^70^.

### Statistical analyses

Statistical analyses of the data were performed using the Dunnett’s test by computing a Student’s t-statistic for each treatment condition compared to the control group (untreated 5xFAD mice) with alpha (α) set to 0.05 using Excel (Microsoft, Redmond, WA). The Dunnett’s test included the non-transgenic WT group when applicable.

## Figures and Tables

**Table 1:** Metabolites with significant differences in AD and WT animals at 3–4 months.

*Frontal Cortex Neurochemical/Cr*	WT (n = 22)	5xFAD (n = 19)	Hedges G; p value
*GABA*	0.4 ± 0.10	0.34 ± 0.05 (−15%)	0.76; p = 0.03
*Glutamate*	1.23 ± 0.143	1.07 ± 0.14 (−13%)	1.46; p = 0.001
*NAA*	0.89 ± 0.13	0.73 ± 0.12 (−18%)	1.24; p = 0.0003
*Aspartate*	0.41 ± 0.1	0.35 ± 0.06 (−14%)	0.69; p = 0.04
*Scyllo-inositol*	0.02 ± 0.007	0.031 ± 0.014 (+ 51%)	0.96; p = 0.007
Ventral Hipp. Neurochemical/Cr	WT (n = 17)	5xFAD (n = 22)	Hedges G; p value
*GABA*	0.38 ± 0.04	0.34 ± 0.06 (−10%)	0.78; p = 0.05
*Glutamate*	0.93 ± 0.10	0.94 ± 0.17	NS
*NAA*	0.53 ± 0.05	0.47 ± 0.08 (−11%)	0.86; p = 0.02
*Aspartate*	0.34 ± 0.04	0.31 ± 0.05	NS
*Scyllo-inositol*	0.028 ± 0.011	0.042 ± 0.017 (+ 50%)	0.91; p = 0.005

Student t-test for unpaired data with unequal variance

## Data Availability

The datasets used and/or analysed during the current study are available from the corresponding author on reasonable request
